# Reporting quality of abstracts in phase III clinical trials of systemic therapy in metastatic solid malignancies

**DOI:** 10.1186/s13063-015-0885-9

**Published:** 2015-08-08

**Authors:** Shanthi Sivendran, Kristina Newport, Michael Horst, Adam Albert, Matthew D. Galsky

**Affiliations:** Ann B. Barshinger Cancer Institute, Lancaster General Health, Lancaster, PA 17604 USA; Hospice and Community Care, Lancaster, PA USA; Research Institute, Lancaster General Health, Lancaster, PA USA; Department of Internal Medicine, Veterans Administration Medical Center, Lebanon, PA USA; Icahn School of Medicine, Tisch Cancer Institute, Mount Sinai, NY USA

**Keywords:** CONSORT, Abstract, Quality, Cancer, Clinical trials, Open access

## Abstract

**Background:**

Manuscript abstracts represent a critical source of information for oncology practitioners. Practitioners may utilize the information contained in abstracts as a basis for treatment decisions particularly when full-text articles are not accessible. In 2007, the Consolidated Standards of Reporting Trials (CONSORT) extension statement for abstracts provided a minimum list of elements that should be included in abstracts. In this study we evaluate the degree of adherence to these recommendations and accessibility of full text publications in oncology publications.

**Methods:**

A systematic review of abstracts of randomized, controlled, phase III trials in metastatic solid malignancies published between January 2009 and December 2011 in PubMed, Medline, and Embase was completed. Abstracts were assigned a completeness score of 0–18 based on the number of CONSORT-recommended elements. Accessibility through open access was recorded.

**Results:**

174 abstracts with data for 95,956 patients were reviewed. The median completeness score was 9 (range, 3–17). Open access to full text articles was available for 80 % of abstracts. The remaining 20 % (35 out of 174) had a median cost of 38 USD (range: $22–49.95). The *least* frequently reported elements were: trial design description (20 %), participant allocation method (13 %), blinding (24 %), trial enrollment status (22 %), registration and name of trial (26 %) and funding source (18 %). The *most* frequently reported elements were eligibility criteria (98 %), study interventions (100 %), and primary endpoint (87 %).

**Conclusion:**

There is poor adherence to the CONSORT recommendations for abstract reporting in publications of randomized cancer clinical trials which could negatively impact clinical decision-making. Full-text articles are frequently available through open access.

## Background

Clinicians utilize peer-reviewed publications to guide the management of patients with cancer. Clinical trial publications provide clinicians with an understanding of the specific patient population that was studied, the details of the experimental intervention, and the potential harms and benefits. Given the importance placed on clinical trials, guidelines, such as those generated by the Consolidated Standards of Reporting Trials (CONSORT) group, have been adopted by the International Committee of Medical Journal Editors to promote comprehensive and transparent reporting [1, 2].

An abstract is a concise summary of a clinical trial that is frequently utilized by clinicians to decide whether or not a study is of sufficient interest to read the full text. For many clinicians, such as those practicing in community-based settings or low and middle income countries, financial and/or information technology barriers limit access to full-text publications and the publically available abstract may be the only source of clinical trial information. However, several analyses have demonstrated inconsistencies in the data presented in abstracts, as compared with full-text manuscripts, that could potentially impact interpretation of the study results [3–9]. Similar inconsistencies have been reported when comparing abstracts presented at scientific meetings with the final study results published in peer-reviewed journals [10–14]. To address these discrepancies, the CONSORT group published an extension statement in 2008 concerning the quality of reporting in journal and conference abstracts. A 17 item checklist with explanations and examples was generated to establish uniform standards of abstract reporting [15]. In this analysis we assess the degree to which oncology clinical trial abstracts adhere to these reporting recommendations.

## Methods

### Literature search

Citations were reviewed from Medline, PubMed, and Embase published between 1 January 2009 and 31 December 2011 to identify eligible publications for analysis. ‘Metastatic” or ‘advanced” were the key words included in the search and were further refined using the following filters: ‘publication dates = 1/1/2009-12/31/2011’; ‘subjects = cancer’; ‘article type = clinical trial, phase III’; ‘language = English’; and ‘species = human.’ Publications that included patients with metastatic solid tumors receiving a pharmacologic intervention in a phase III clinical trial, as indicated by study authors, were included in the analysis. All other studies, including phase I and II studies, observational studies, letters, case reports, editorials, and studies exploring a device or behavioral intervention, were excluded. In the setting where multiple publications were found from the same trial, only the initial publication was used for the analysis. If the initial publication was published prior to 2009, neither that publication nor subsequent related publications were included in the analysis.

### Data extraction

The CONSORT extension for abstract reporting outlines 17 general recommendations (Table 1) [15]. Additional recommendations are found in an accompanying paper entitled ‘CONSORT for reporting randomized controlled trials in journal and conference abstracts: explanation and elaboration.’ A multidisciplinary panel of five providers reviewed these recommendations and assembled a consensus list of 18 key reporting elements that were used in data collection and outlined in Table 1. The reporting element ‘Eligibility criteria for participants and the settings where the data were collected’ was divided into two separate reporting elements - ‘Eligibility criteria for participants’ and ‘The settings where the data was collected.’ Allocation concealment was evaluated based on specific criteria provided by the CONSORT committee's explanations and elaborations [15]. One CONSORT recommendation not included was ‘contact details for the corresponding author’ as this is specific to conference abstracts and no conference abstracts were reviewed in our analysis.

**Table 1:** Elements of abstract reporting

Manuscript section	CONSORT recommendations	Elements included in current analysis	Abstract reporting element number
Title	1. Identification of the study as randomized	Identification of the study as randomized	1
Authors	2. Contact details for the corresponding author	Not included in the current analysis^a^	
Trial design	3. Description of the trial design (e.g., parallel, cluster, non-inferiority)	Abstract specifies whether specific description of the trial design was included (e.g., parallel, cluster, non-inferiority)	2
Methods	4. Eligibility criteria for participants and the settings where the data were collected	Abstract specifies the eligibility criteria for participants relating to demographics, clinical diagnosis, and co morbid conditions.	3
		Abstract specifies the setting in which the trial took place (for example primary, secondary, tertiary centers)	4
	5. Interventions intended for each group	Abstract specifies medication intervention intended for each study group	5
	6. Specific objective or hypothesis	Abstract specifies the objective or hypothesis of the study	6
	7. Clearly defined primary outcome for this report	Abstract clearly states the primary outcome or endpoint in the study	7
		Abstract describes over what period of time the primary outcome or endpoint was assessed	8
	8. How participants were allocated to interventions	Abstract describes the method by which participants were assigned to interventions to ensure adequate concealment (e.g. use of computer or random number table)	9
	9. Whether or not participants, care givers, and those assessing the outcomes were blinded to group assignment	Abstract specifies whether participants, care givers, and those assessing the outcomes were masked or blinded to the group allocation	10
Results	10. Number of participants randomized to each group	Abstract reports absolute numbers of patients randomized to each group	11
	11. Trial status	Abstract reports the status of the trial and whether it is ongoing, closed to recruitment or closed to follow-up	12
	12. Number of participants analyzed in each group	Abstract reports either absolute numbers analyzed in each group or indicates this is an intention to treat analysis	13
	13. For the primary outcome, a result for each group and the estimated effect size and its precision	Abstract reports trial results as a summary of the outcome for each group and the contrast between the groups (examples include relative risk, odds ratio, hazard ratio, confidence intervals)	14
Harms	14. Important adverse events or side effects	Abstract explicitly describe any important or unexpected adverse events	15
Conclusions	15. General interpretation of results	Abstract states conclusions of the trial consistent with reported results.	16
	16. Registration number and name of trial register	Abstract reports registration number and name of trial register	17
	17. Source of funding	Abstract lists funding source for study	18

^a^Not included in the current analysis as the multidisciplinary panel that reviewed the CONSORT abstract extension statement concluded that this element is specific to conference abstracts

Eligible publications were evaluated for each of the 18 reporting elements in the abstract only. Additional data were extracted from either the abstract or full-text manuscript including the sample size, intervention type, use of placebo control, publication year, funding source, journal name and impact factor, and whether the primary study endpoint was met and specifically commented on in the discussion portion of the abstract. We also investigated whether the manuscript was available in full-text form though open access. If the full text was not available through open access, we recorded the cost (in US dollars) to access the full-text manuscript. Finally, we recorded whether the article was published in a journal that endorses the CONSORT statement.

The intervention types were classified as targeted therapy, chemotherapy, immunotherapy, or hormonal therapy. The primary endpoint was obtained from either the abstract or full manuscript. The results section was reviewed to determine if the primary endpoint was met. We compared regulatory approval dates derived from the Drugs@FDA Website with the manuscript publication date to assess whether the intervention was approved for another indication. Data extraction was done by two independent reviewers (SS and KN) and discrepancies resolved by a third reviewer (AA). Inter-rater agreement x kappa values ranged from 0.66 to 1.00 with a mean ± standard deviation of 0.88 ± 0.10.

### Statistical analysis

The primary objective of this systematic review was to describe the quality of abstract reporting in phase III randomized controlled trials of systemic therapy for metastatic solid tumors in the context of the CONSORT abstract extension statement. Quality of abstract reporting was defined by the ‘completeness score’, calculated as the total number of CONSORT elements (Table 1) reported in each abstract. Each abstract received a score of 0–18. The secondary objective was to determine which clinical trial characteristics were associated with the ‘completeness score.’ We constructed linear models to assess the unadjusted association between the completeness score and covariates in the abstracts. This facilitated the assessment of categorical and continuous covariates in the same model structure. An exploratory objective was to describe the accessibility of full-text manuscripts through open access. All analyses were performed using Stata version 13.1 (College Station, TX, USA) using two-sided *p* values and *p* <0.05 considered as significant.

## Results and discussion

### Literature search

**Fig. 1:**
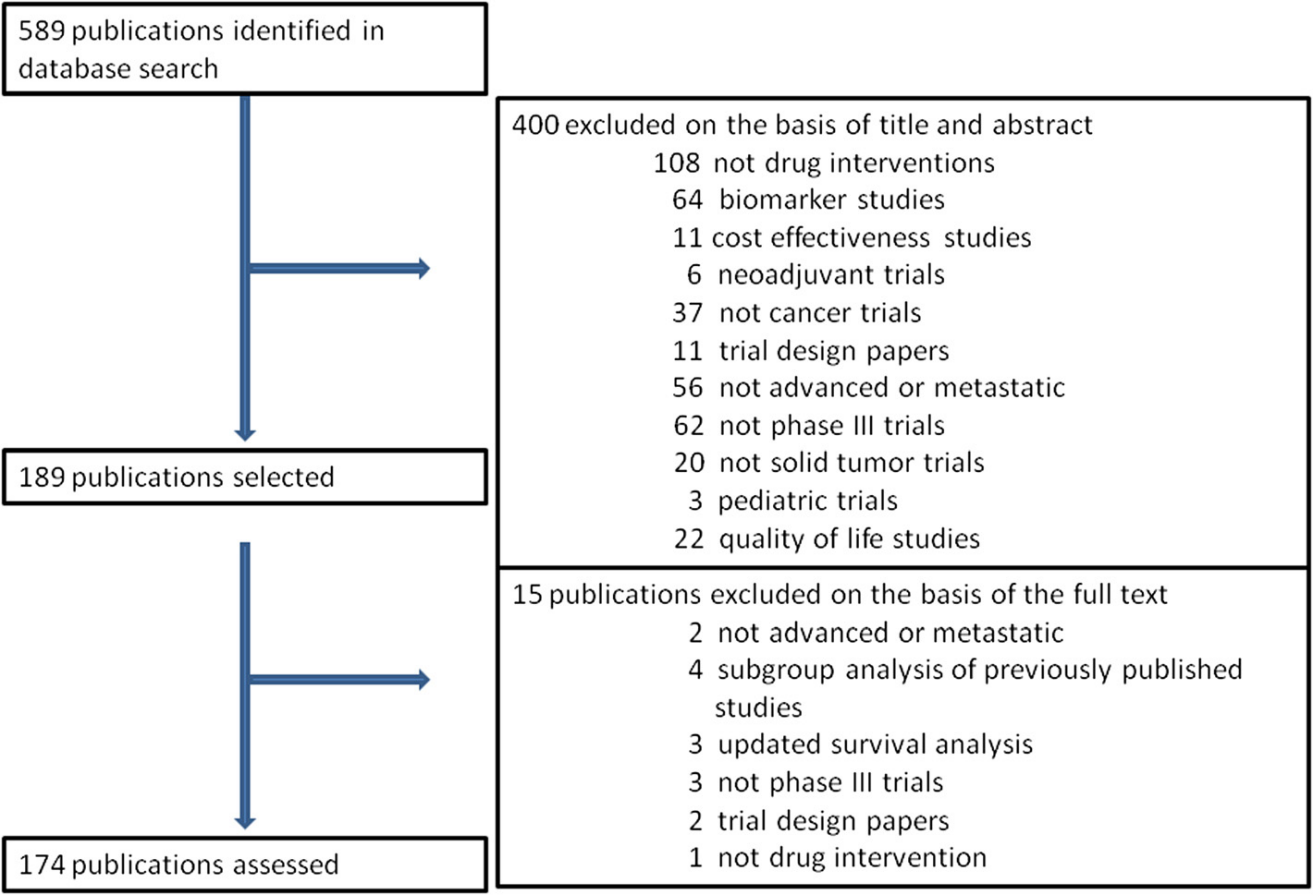
Flowchart of screening of publications included in the analysis

Of the 589 potentially relevant publications identified in our search, 174 publications met the eligibility criteria for inclusion in the analysis. The reasons for study inclusion and exclusion are detailed in Fig. 1.

The characteristics of the publications included in the analysis are detailed in Table 2. A total of 95,956 patients were involved in the trials included in this review with THE median sample size being 421 (range 19–4,312). Breast cancer was the most common cancer subtype explored (21 %) and chemotherapy was the most common intervention (48 %). The majority of studies were supported by industry.

**Table 2:** Characteristics of studies included in analysis (n = 174)

Characteristic	Value
Median sample size (range)	421 (19–4,312)
Placebo-controlled	45 (26 %)
Intervention type	
Chemotherapy	83 (48 %)
Hormonal therapy	7 (4 %)
Targeted therapy	60 (34 %)
Immunotherapy	11 (6 %)
Chemotherapy + targeted therapy	7 (4 %)
Chemotherapy + immunotherapy	6 (3 %)
Trial met primary endpoint	75 (43 %)
Funding source	
Industry	112 (64 %)
Government	16 (9 %)
Industry and Government	14 (8 %)
Other	9 (5 %)
Funding source not reported	22 (13 %)
No funding	1 (<1 %)
Experimental drug approved for other indication	132 (76 %)
Year of publication	
2009	49 (28 %)
2010	58 (33 %)
2011	67 (39 %)
Cancer type	
Breast	37 (21 %)
Colorectal	19 (11 %)
Gastric or gastroesophageal	8 (5 %)
Head and neck	5 (3 %)
Lung	37 (21 %)
Melanoma	9 (5 %)
Ovarian	12 (7 %)
Pancreas	11 (6 %)
Prostate	9 (5 %)
Renal	8 (5 %)
Other	19 (11 %)
Journal	
Annals of Oncology	16 (9 %)
Breast Cancer Research and Treatment	6 (3 %)
British Journal of Cancer	3 (2 %)
Cancer	5 (3 %)
European Journal of Cancer	10 (6 %)
Journal of Clinical Oncology	60 (34 %)
Lancet	11 (6 %)
Lancet Oncology	12 (7 %)
New England Journal of Medicine	14 (8 %)
Other	37 (21 %)
Impact factor of journals, median (range)	18 (1–53)

Results are presented as number (%) unless stated otherwise

### Abstract reporting

The proportion of abstracts reporting each of the 18 reporting elements is shown in Table 3. The majority of the abstracts did not provide a specific description of the trial design (20 %) or the setting in which the trial took place (14 %). The method by which participants were assigned to interventions to ensure concealment was only reported in 13 % of abstracts and masking or blinding of the group allocation was only reported in 24 % of abstracts. Most abstracts did not report the time period over which the primary outcome was assessed (17 %) or how many patients were analyzed in each group (26 %).

**Table 3:** Proportion of articles addressing each of the 18 abstract reporting elements (n = 174)

Reporting elements	Number (%)
1. Identification of the study as randomized	100 (57 %)
2. Abstract specifies whether specific description of the trial design was included (e.g., parallel, cluster, non-inferiority)	34 (20 %)
3. Abstract specifies the eligibility criteria for participants relating to demographics, clinical diagnosis, and comorbid conditions.	171 (98 %)
4. Abstract specifies the setting in which the trial took place (e.e., primary, secondary, tertiary centers)	24 (14 %)
5. Abstract specifies medication intervention intended for each study group	174 (100 %)
6. Abstract specifies the objective or hypothesis of the study	166 (95 %)
7. Abstract clearly states the primary outcome or endpoint in the study	151 (87 %)
8. Abstract describes over what period of time the primary outcome or endpoint was assessed	29 (17 %)
9. Abstract describes the method by which participants were assigned to interventions to ensure adequate concealment (e.g., use of computer or random number table)	22 (13 %)
10. Abstract specifies whether participants, care givers, and those assessing the outcomes were masked or blinded to the group allocation	42 (24 %)
11. Abstract reports absolute numbers of patients randomized to each group	80 (46 %)
12. Abstract reports the status of the trial and whether it is ongoing, closed to recruitment or closed to follow up	39 (22 %)
13. Abstract reports either absolute numbers analyzed in each group or indicates this is an intention-to-treat analysis	45 (26 %)
14. Abstract reports trial results as a summary of the outcome for each group and the contrast between the groups (examples include relative risk, odds ratio, hazard ratio, confidence intervals)	152 (87 %)
15. Abstract explicitly describes any important or unexpected adverse events	129 (74 %)
16. Abstract states conclusions of the trial consistent with reported results.	168 (97 %)
17. Abstract reports registration number and name of trial register	46 (26 %)
18. Abstract lists funding source for study	31 (18 %)

Some reporting elements were applied more consistently across abstracts. All of the abstracts analyzed reported the intervention intended for each study group (100 %). The objective or hypothesis was reported in 95 % of abstracts and the primary outcome was reported in 87 % of abstracts. Summary statistics for the outcome for each group (87 %) and conclusions based on the results reported in the abstract (95 %) were reported consistently. Important or unexpected adverse events were reported in 74 % of abstracts.

### Abstract reporting completeness scores

**Fig. 2:**
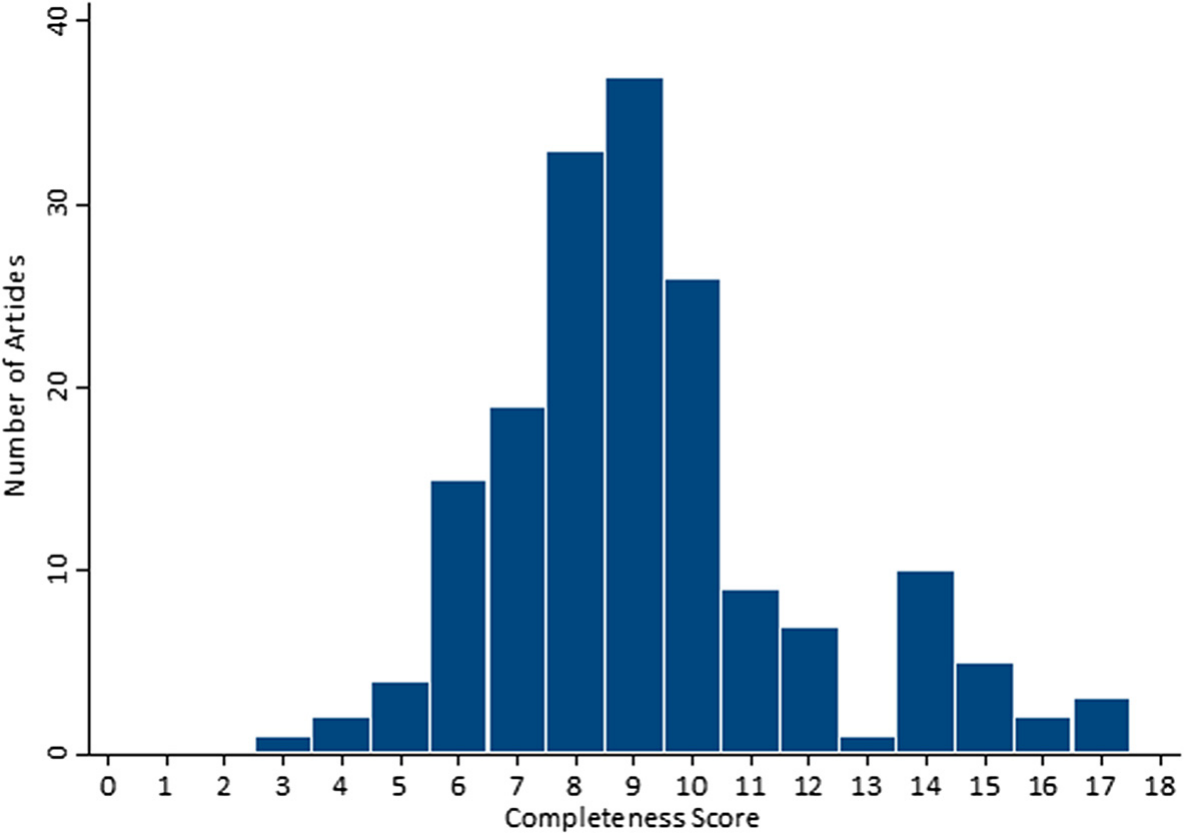
Distribution of abstract reporting completeness scores (n = 174). The completeness score was derived by adding each of the 18 abstract reporting elements addressed in each abstract

The mean ± standard deviation completeness score was 9.2 ± 2.7 (range 3–17; Fig. 2). Among 174 abstracts analyzed, 144 (83 %) were published in journals that endorse the CONSORT recommendations. There was no significant difference in completeness scores between abstracts published in journals that do (9.3 ± 2.8), and do not (8.7 ± 2.1) endorse the CONSORT recommendations (*p* = 0.241). There were also no significant differences in the mean completeness scores for placebo-controlled trials, treatment type or funding source (see Table 4). We did find differences (*p* = 0.014) in the mean completeness score for studies that had interventions approved for another indication (8.9 ± 2.6) having lower completeness scores than for studies with interventions not approved for another indication (10.1 ± 2.9). If the trial met the efficacy endpoint, the mean completeness score was higher (9.8 ± 3.0) than if the trial did not meet the efficacy endpoint (8.8 ± 2.4) (*p* = 0.018). We also found a linear trend with increasing completeness scores based on the year of publication across 2009, 2010 and 2011 (*p* = 0.039).

**Table 4:** Publication characteristics associated with abstract reporting completeness score

Publication characteristics	Number	Completeness score mean ± SD	*P* value
Endorsement of CONSORT recommendations			0.241
No	30	8.7 ± 2.1	
Yes	144	9.3 ± 2.9	
Placebo controlled			0.532
No	129	9.3 ± 2.7	
Yes	44	9.0 ± 2.6	
Treatment type			0.789
Chemotherapy	83	9.1 ± 2.8	
Targeted	60	9.4 ± 2.7	
Other^a^	31	9.2 ± 2.5	
Funding source			0.729
Industry	112	9.3 ± 2.7	
Other	62	9.1 ± 2.6	
Intervention approved for another indication			0.014
No	43	10.1 ± 2.9	
Yes	131	8.9 ± 2.6	
Year of publication			0.039
2009	49	8.6 ± 2.7	
2010	58	9.0 ± 2.4	
2011	67	9.9 ± 2.8	
Trial met efficacy endpoint			0.018
No	99	8.8 ± 2.4	
Yes	75	9.8 ± 3.0	
Open access (available free)			0.210
No	35	8.7 ± 2.3	
Yes	139	9.4 ± 2.8	

^a^Other treatment type includes chemotherapy and immunotherapy, chemotherapy and targeted, hormonal, and immunotherapy

### Accessibility of full-text manuscripts through open access

Of the 174 abstracts reviewed for our analysis, the accompanying full text of the manuscript was available through open access in 80 % (139/174). There was no significant difference in the mean completeness scores for publications that were available free of charge versus those that required purchase (9.4 ± 2.8 versus 8.7 ± 2.3 respectively; *p* = 0.210). The median cost to purchase an article not available through open access was 38 US dollars (range: $22–$49.95).

## Conclusion

An abstract is a snapshot of the overall design and results of a clinical trial and in many circumstances may be the only portion of a manuscript read by a clinician due to time constraints and barriers to accessing full-text publications. Therefore, sufficiently detailed and transparent reporting of oncology clinical trial abstracts is critical. Several guidelines have been developed, including those from the CONSORT group, in an effort to standardize the quality of abstract reporting [1, 2, 4, 15]. However, despite the 2008 CONSORT extension statement for reporting randomized controlled trials in journal and conference abstracts, there is significant heterogeneity in reporting of individual elements as demonstrated by our analysis and that of others [16–23].

Variability in reporting randomized controlled trials in journal abstracts may be secondary to several factors. Authors may be unaware of the CONSORT extension recommendations and journals may not require or enforce compliance to these guidelines. The CONSORT extension guidelines are not oncology specific and emphasis on certain reporting elements may be different for various disciplines. Limitations on word count for abstracts may also impact the ability of authors to comply with the CONSORT recommendations.

Notably, our unadjusted analyses demonstrated that most analyzed variables, including journal endorsement of CONSORT recommendations, placebo controlled trials, treatment type, or funding source, were not associated with completeness of abstract reporting. This suggests that suboptimal reporting is not limited to subsets of journals but rather a generalized issue. Interestingly, we reported that completeness scores have been linearly increasing over time as publications from 2011 had higher completeness scores. Our inclusion of publications spanning only a 3 year time interval (2009–2011), however, limits the interpretation of this finding.

There are several prior analyses addressing the quality of abstract reporting in randomized clinical trials but the majority of these have not evaluated oncology trials. However, during the preparation of this manuscript, Ghimire et al. published an analysis of oncology randomized controlled trials over a three-year period both prior to and after adoption of the CONSORT extension statement for abstract reporting. Substantial heterogeneity and selectivity in the quality of abstract reporting was demonstrated with only half of the CONSORT recommendations consistently being reported after the adoption of the CONSORT extension statement [24]. Our analysis extends these findings in several ways. We investigated the potential association between several study characteristics and abstract reporting completeness. For example, we demonstrated that journal endorsement of the CONSORT statement was not associated with abstract reporting completely, nor was public availability of the full-text manuscript. These findings suggest that other factors, including authors’ lack of awareness of the guidelines or journals lack of enforcement of guidelines, may potentially be contributing to the poor quality of abstract reporting. Another study done prior to adoption of the CONSORT extension statement utilized a checklist derived from the original CONSORT statement and applied to American Society of Clinical Oncology Annual Meeting abstracts and also demonstrated the generally poor quality of abstract reporting.

Prior studies have investigated the use of a more stringent application of the CONSORT guidelines during the peer review process in improving the quality of abstracts. Hopewell et al. conducted an interrupted time series analysis of the quality of abstracts describing medical randomized controlled trials both before and after the implementation of the CONSORT extension statement for abstracts in five leading medical journals. Journals were divided into three categories - those that did not endorse the CONSORT guidelines, those that endorsed the CONSORT guidelines but did not have an active policy to enforce them, and those that endorsed the CONSORT guidelines and had an active editorial process to enforce them. Interestingly, there was only a significant change in the mean number of items reported pre and post implementation of the CONSORT extension statement in the journals that had an editorial process to enforce the guidelines during the peer review process [25]. This highlights the importance of not only awareness of the guidelines by authors but the need for implementation and enforcement of these guidelines during the editorial review. Cobo et al. investigated the effect of adding an additional review based on reporting guidelines to the conventional peer review process for all original research manuscripts submitted to one journal. The overall quality of the manuscripts was improved with the addition of a second guideline-based review. Interestingly, during the editing process, authors were more likely to make manuscript revisions based on the conventional reviews rather than the guideline-based reviews. The authors postulated several potential reasons for this including a lack of awareness of these guidelines when designing a research study, making it more difficult to adhere to reporting certain elements in the publication process [26].

Accurate and transparent reporting of clinical trial information in abstracts may be particularly important in situations in which there is no access to full-text articles. Community oncologists, who provide 85 % of the cancer care in the USA, as estimated by the National Cancer Institute, are less likely to have comprehensive journal access and may be less likely to read multiple full-text articles when payment is required. The cost of purchasing these articles may be prohibitive to oncology physicians in the developing world where cost and access to information technology is potentially prohibitive, and who may rely only on the abstract content to make clinical decisions. Fortunately, we did find that open access to full-text articles was available in 80 % of our dataset. It is arguably more imperative for conference abstracts, in which a full text of a research study is not yet available, to stringently follow CONSORT guidelines as this is the only reflection of the details of a study.

There are some limitations to our study. This analysis was conducted specifically in randomized phase III metastatic solid tumor trials and further standardization of reporting may be necessary for other types of trials and disease states. The analysis included only English language studies. The importance of certain reporting elements may not be agreed upon universally by all authors or sub-specialities. Some may argue that several of the most poorly reported elements such as the setting in which the trial took place, reporting of allocation concealment, recruitment status, registration number, and funding source are important elements to include in the full text but are not necessarily informative in the abstract. Removal of these may raise completeness scores as well as allow researchers more word space to address other key trial details in the abstract. Future studies are needed to address this.

Importantly, CONSORT guidelines and extension statements represent a minimum standard in reporting. Adoption and expansion of these guidelines by the oncology community, and implementation and enforcement of these guidelines by journal editors, will potentially lead to greater adherence and improved quality of publications and improved clinical decision making.

## Abbreviations

CONSORT: Consolidated Standards of Reporting Trials
